# The Natural History and Prognostic Determinants of Untreated Hepatocellular Carcinoma in a Sub-Saharan African Cohort

**DOI:** 10.1007/s12029-026-01414-0

**Published:** 2026-02-20

**Authors:** Sanju Sobnach, Urda Kotze, C. Wendy Spearman, Mark W. Sonderup, Inae Kim, Keith Venter, René Krause, Muhammad Emmamally, Marc Bernon, Tinus du Toit, Luiz F. Zerbini, Eduard Jonas

**Affiliations:** 1 Surgical Gastroenterology Unit, Division of General Surgery, Department of Surgery, Faculty of Health Sciences, University of Cape Town, Cape Town, South Africa; 2 Division of Hepatology, Department of Medicine, Faculty of Health Sciences, University of Cape Town, Cape Town, South Africa; 3 Division of Interdisciplinary Palliative Care and Medicine, Department of Community, Family and Emergency Care, Faculty of Health Sciences, University of Cape Town, Cape Town, South Africa; 4 Transplant Unit, Division of General Surgery, Department of Surgery, Faculty of Health Sciences, University of Cape Town, Cape Town, South Africa; 5 International Centre for Genetic Engineering and Biotechnology (ICGEB), Cape Town, South Africa; 6 Division of General Surgery, Department of Surgery, Faculty of Health Sciences, University of Cape Town, Cape Town, South Africa

**Keywords:** Hepatocellular carcinoma, Untreated, Palliative care, Sub-Saharan africa, Prognostic factors, Natural history, Hepatitis b virus

## Abstract

**Background:**

Data on the natural history of untreated hepatocellular carcinoma (HCC) are limited and derive mostly from patients in untreated arms of randomised control trials, conducted in high income countries. In this study, we determined the natural history of untreated HCC and identify predictors of survival in a predominantly SSA cohort of patients managed at Groote Schuur Hospital, Cape Town, South Africa.

**Methods:**

A 35-year retrospective cohort study of 469 patients with untreated HCC managed at Groote Schuur Hospital, Cape Town, South Africa from 1990 to 2025 was conducted. Demographics, clinical features, laboratory data, radiological findings and survival were analysed. Multivariate Cox regression identified independent predictors of mortality.

**Results:**

The cohort comprised 347 (74%) men, with a median age of 48 [19–89] years. The majority (380/469, 81.0%) were from South Africa, while the remaining 89 patients originated mostly from other neighbouring SSA countries. Chronic hepatitis B virus (HBV) infection was the leading aetiology (53.3%). Most (96.3%) presented with advanced disease (BCLC stage C or D), with high rates of multifocal tumours (69.1%), portal vein tumour thrombosis (38.8%) and extrahepatic metastases (32.6%). The median overall survival was 36.5 days and the one, six and 12-month survival rates were 59.8%, 14.4% and 7.6% respectively. Independent predictors of mortality included poor performance status, elevated alpha-fetoprotein levels, hypoalbuminemia, higher CTP grade, elevated MELD-Na and advanced BCLC stage.

**Conclusion:**

Untreated HCC in sub-Saharan Africa is marked by advanced presentation and aggressive disease with rapid clinical deterioration and poor long-term survival. This study highlights the critical need for earlier diagnosis, HBV prevention and the development of accessible, early context-appropriate palliative care interventions in resource-limited settings.

## Key Points

• In a sub-Saharan cohort, most patients with untreated hepatocellular carcinoma present with advanced-stage disease.

• Chronic hepatitis B virus infection is the predominant aetiology and associated with multifocal tumours, macrovascular invasion and extrahepatic metastases.

• In patients who receive best supportive care only, there is rapid clinical deterioration and very poor long-term survival.

**Graphical Abstract:**

## Graphical abstract

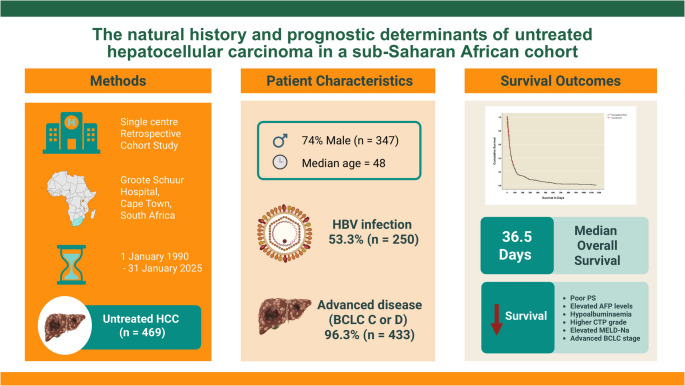

## Background

Hepatocellular carcinoma (HCC) represents a global public health challenge [1]. Despite significant progress in the management of HCC over the past decade, its incidence-to-mortality ratio approaches 1.0, highlighting the disease’s fatality [2–5]. The global burden of HCC continues to rise, with the highest prevalence reported in sub-Saharan Africa (SSA) and southeast Asia [1, 2, 6–8]. The annual HCC-related mortality is projected to reach 1.4 million by 2040 [9]. 

In SSA, HCC is mainly caused by chronic hepatitis B virus (HBV) infection, and frequently presents with multifocal tumours, extrahepatic metastases and macrovascular invasion (portal vein tumour thrombosis [PVTT] and/or hepatic vein tumour thrombosis [HVTT]) [6, 7, 10–12]. Consequently, the majority (84%) of patients receive best supportive care (BSC) only with almost no survivors at one year [7]. In high income countries (HICs), 40% of patients are treated with curative-intended therapies (ablation, resection, liver transplantation), resulting in five-year survival rates surpassing 70% [5, 8, 13, 14]. 

Despite marked disparities in access to curative-intended therapies between SSA and HICs, a paradoxically substantial proportion of HCC patients in both regions receive BSC only [2, 5, 7, 10, 12, 14–20]. Data from the Veterans Administration centers and Surveillance, Epidemiology and End Results (SEER)-Medicare indicate that between 24% and 60% of HCC patients in the United States do not receive any form of treatment [17, 18]. In countries with universal healthcare, up to a third of patients receive BSC only. An analysis of 63 668 HCC cases in South Korea found that 27.6% were untreated [16]. Of the 7325 patients with HCC in Sweden between 2009 and 2023, 2466 (33.7%) did not receive any life-prolonging treatments including transarterial chemotherapy (TACE) and systemic therapies [21]. 

Survival in patients with HCC is heterogeneous and varies across geographical regions, largely reflecting differences in underlying disease aetiology, biology and healthcare accessibility [3, 7, 10, 15, 16, 18–20, 22–34]. The natural history of untreated HCC and its associated prognostic factors have not previously been characterised in SSA. Such data are essential in contextualising the survival benefits of other palliative therapies including TACE and systemic therapies such as sorafenib, lenvatinib, atezolizumab/bevacizumab, durvalumab/tremelimumab, regorafenib, nivolumab or pembrolizumab in patients with advanced HCC, who constitute the predominant disease burden in SSA [6, 7, 12, 35, 36]. Given the socio-economic constraints and public health challenges in SSA, the implementation of these palliative therapies will not only require robust evidence of survival benefit in local populations, but also demonstrable survival and quality of life outcomes that significantly exceed the natural history of untreated HCC [37–39]. 

The natural history of untreated patients is also essential for evaluating the validity of biological and radiological prognostic markers in HCC, adjusting for confounding factors in observational research and informing sample size calculation and patient stratification in future randomised controlled trials (RCTs). In this study, we will determine the natural history of untreated HCC and identify predictors of survival in a predominantly SSA cohort of patients managed at Groote Schuur Hospital, Cape Town, South Africa.

## Methods

### Study Design and Ethical Approval

We conducted a single-center, retrospective cohort study of patients with untreated HCC (received upfront BSC with no other treatments) at Groote Schuur Hospital, Cape Town, South Africa, from 1 January 1990 to 31 January 2025. Ethical approval was granted by the institutional review board of the Faculty of Health Sciences at the University of Cape Town (IRB00001938, HREC REF 331/2025). The data for this study were extracted from two HCC registries at Groote Schuur Hospital. The first registry, a paper-based system, included data for patients treated from 1 January 1990 to 31 December 2016. From January 2017 onward, patient data were prospectively entered into an electronic registry using a faculty-secure REDCap (Research Electronic Data Capture) platform hosted by the University of Cape Town (IRB00001938, R003/2019) [40]. 

## Inclusion and Exclusion Criteria

Patients eligible for inclusion were aged 18 years or older, with a confirmed diagnosis of HCC based on either radiological or histological criteria [13, 41]. A diagnosis of fibrolamellar HCC or mixed cholangio-HCC was considered exclusionary.

## Data Collection

The following data were extracted: patient demographics, performance status (PS) as per World Health Organisation (WHO) or Eastern Cooperative Oncology Group (ECOG) criteria, presenting symptoms and duration thereof, clinical examination findings, comorbidities and lifestyle factors including alcohol consumption and smoking history. Laboratory data included full blood count, international normalised ratio (INR), renal and liver function tests, serum alpha-fetoprotein (AFP) levels and HBV, hepatitis C virus (HCV) and human immunodeficiency virus (HIV) serology. Cirrhosis status was classified using either histopathology (when available) or well-documented clinical and radiological features consistent with cirrhosis/portal hypertension, including a nodular liver contour, splenomegaly, varices and/or ascites. Where the medical record and imaging reports lacked sufficient detail to support classification, the cirrhosis status was recorded as unknown.

Abdominal ultrasound (US) and contrast-enhanced (CE) cross-sectional imaging (computed tomography (CT) and magnetic resonance imaging (MRI)) reports were reviewed. We also documented whether the diagnosis was made through surveillance or at the time of presentation with symptoms. The Child Turcotte Pugh score (CPS), Model for end stage liver disease-sodium (MELD-Na) score and Barcelona Clinic Liver Cancer (BCLC) stage were calculated and recorded. Finally, survival outcomes were recorded and analysed.

### Statistical Analysis

Categorical variables were presented as frequencies and percentages and comparisons were made using the chi-square test or Fisher’s exact test. Continuous variables were expressed as medians and the Student’s t-test or the Wilcoxon rank sum test used for comparisons. Survival curves were generated using the Kaplan-Meier method and compared using the log-rank test. Factors associated with OS were determined using univariate and multivariate Cox proportional hazards regression models. Only variables with univariate significance were entered into a multivariate Cox model. Variables with the highest p-value were removed from the model using backward stepwise elimination until all variables in the model remained statistically significant. Statistical significance was based on a two-sided test at 5%.

## Results

Over the 35-year study period, a total of 728 patients with HCC were managed at Groote Schuur Hospital. Of these, 469 (64.4%) received BSC only and constituted the untreated HCC cohort. The latter comprised 347 (74%) men, with a median age of 48 [19–89] years. The majority (380/469, 81.0%) were from South Africa, while the remaining originated mostly from other SSA countries (Table 1). A PS of 3 and 4 was recorded in 122 (26.8%) and 31 (6.8%) patients, respectively. Hepatitis B virus infection was present in more than half of the cohort (250/469, 53.3%) and only 17 (3.6%) tested positive for HCV. The median duration of symptoms was 60 (range: 1-1500) days, with pain (74.8%) and weight loss (62.5%) being the most frequently reported. On clinical examination, hepatomegaly (54.6%) and ascites (48.4%) were the most observed findings. Child Turcotte Pugh scores were available in 453 patients, of whom 154 (34%), 193 (42.6%) and 106 (23.4%) had grade A, B and C disease, respectively. The BCLC stage was documented in 450 patients, with the majority presenting with advanced disease: 246 (54.7%) were classified as BCLC Stage C and 187 (41.6%) as stage D HCC. The median MELD-Na was 14 (range:6–38) and available in 444 patients.

**Table 1:** Characteristics of 469 patients with untreated hepatocellular carcinoma. WHO = World Health Organisation, ECOG = Eastern Cooperative Oncology Group, PS = Performance Status, HBV=hepatitis B virus, HIV = Human immunodeficiency virus, HCV=hepatitis C virus, AST = Aspartate aminotransferase, ALT = Alanine transaminase, GGT = Gamma-glutamyltransferase, ALP = Alkaline phosphatase, MELD-Na: Model for End-Stage Liver Disease, ^§^Reported in 455 patients, ^§§^ Reported in 453 patients, *Reported in 444 patients, Other: Portugal = 1, Taiwan = 1, China = 1, Bangladesh = 3

Patient characteristics	n=469
Age (years )	48 (19-89)
Sex	
Male	347 (74%)
Female	122 (26%)
Country of origin	
South Africa	380 (81%)
Malawi	31 (6.6%)
Zimbabwe	27 (5.8%)
Democratic Republic of Congo	10 (2.1%)
Mozambique	3 (0.6%)
Namibia	2 (0.4%)
Nigeria	2 (0.4%)
Somalia	2 (0.4%)
Zambia	2 (0.4%)
Kenya	1(0.2%)
Rwanda	1(0.2%)
Tanzania	1 (0.2%)
Other^#^	6 (1.3%)
WHO/ECOG PS^§^	
0	15 (3.3%)
1	143 (31.4%)
2	144 (31.6%)
3	122 (26.8%)
4	31 (6.8%)
Comorbidities	
Diabetes mellitus	46 (9.8%)
Hypertension	79 (16.8%)
Human immunodeficiency virus	62 (13.2%)
HBV infection	250 (53.3%)
HIV-HBV coinfection	53 (11.3%)
HCV infection	17 (3.6%)
Schistosomiasis	12 (2.6%)
Duration of symptoms (days)	60 (0-1500)
Symptoms	
Pain	351 (74.8%)
Weight loss	293 (62.5%)
Anorexia	134 (28.6%)
Vomiting	102 (21.7%)
Nausea	88 (18.8%)
Gastrointestinal Bleeding	38 (8.1%)
Fever	25 (5.3%)
Signs	
Hepatomegaly	256 (54.6%)
Ascites	227 (48.4%)
Palpable mass	142 (30.3%)
Jaundice	125 (26.7%)
Hepatic encephalopathy	28 (6%)
Haemoglobin (g/dL)	11.4 (4.6-20.4)
Platelet count (×10^9^/L)	267 (29-982)
International normalised ratio	1.3 (0.9-4)
Total bilirubin (µmol/L)	25 (3-758)
Conjugated bilirubin (µmol/L)	14 (1-501)
AST (U/L)	143 (12-7999)
ALT (U/L)	56 (5-1054)
GGT (U/L)	321 (4-2531)
ALP (U/L)	2596 (12-5180)
Creatinine (µmol/L)	69 (8-538)
Sodium (mmol/L)	135 (112-149)
Albumin (g/L)	32 (12-86)
Child Turcotte Pugh Grade^§§^	
A	154 (34%)
B	193 (42.6%)
C	106 (23.4%)
Barcelona Clinic Liver Cancer Stage	
0	0 (0%)
A	3 (4.2%)
B	3 (4.2%)
C	34 (47.9%)
D	31 (43.6%)
MELD-Na score*	14 (6-38)

Underlying liver cirrhosis was present in 213 (51.7%) patients and absent in 199 (48.3%) patients. There were not enough data to determine the presence or absence of cirrhosis in 57 patients. In patients with liver cirrhosis, chronic HBV infection was the aetiology of HCC in 57.3% while in the non-cirrhotic group, 54.3% had HBV-related HCC. Of the remaining 385, 266 (69.1%) had multifocal disease. The median AFP was 3613 (range:0.1-2721870) µg/L. Portal vein tumour thrombosis and HVTT were seen in 182 (38.8%) and 72 (16.2%) of the patients, respectively. Upon presentation, 153 (32.6%) had extrahepatic metastases with lungs being the most common site (93/153, 60.8%) (Table 2).

**Table 2:** Tumour characteristics in 469 patients with untreated hepatocellular carcinoma. Data are reported as n (%) or median (IQR). PVTT=Portal vein tumour thrombosis, HVTT=Hepatic vein tumour thrombosis, ^§^Reported in 385 patients

Tumour characteristics	n=469
Serum alpha-fetoprotein (µg/L)	3613.5 (0.1-2721870)
Background liver	
Cirrhotic	213 (45.4%)
Non-cirrhotic	199 (42.4%)
Unknown	57 (12.2%)
Number of lesions^§^	
1	119 (30.9%)
≥ 2	266 (69.1%)
Macrovascular invasion	
Any PVTT	182 (38.8%)
Any HVTT	72 (16.2%)
Extra-hepatic metastases	
Lung	93 (60.8%)
Bone	17 (11.1%)
Lymph Nodes	18 (11.8%)
CNS	15 (9.8%)
Peritoneal	9 (5.9%)

**Fig. 1:**
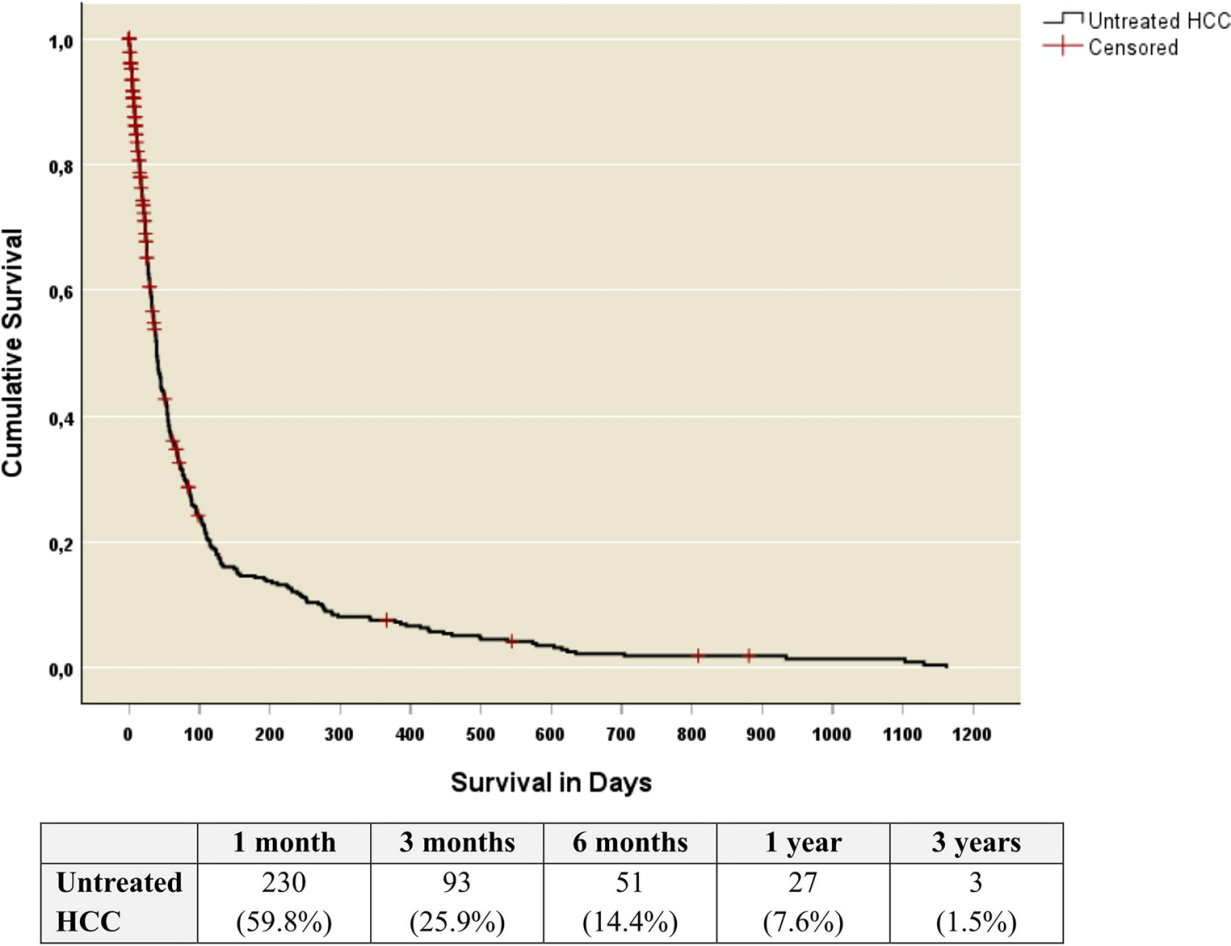
Kaplan Meier indicating the cumulative survival of untreated hepatocellular carcinoma (HCC) patients

Eighty-three (17.7%) patients were lost to follow up. At the end of the study period, only four (0.9%) patients were alive and the median survival of patients with untreated HCC was 36.5 (range:0-1162) days. The one-month, six-month, one-year and three-year survival rates were 59.8%, 14.4%, 7.6% and 1.5%, respectively (Fig. 1).

**Fig. 2:**
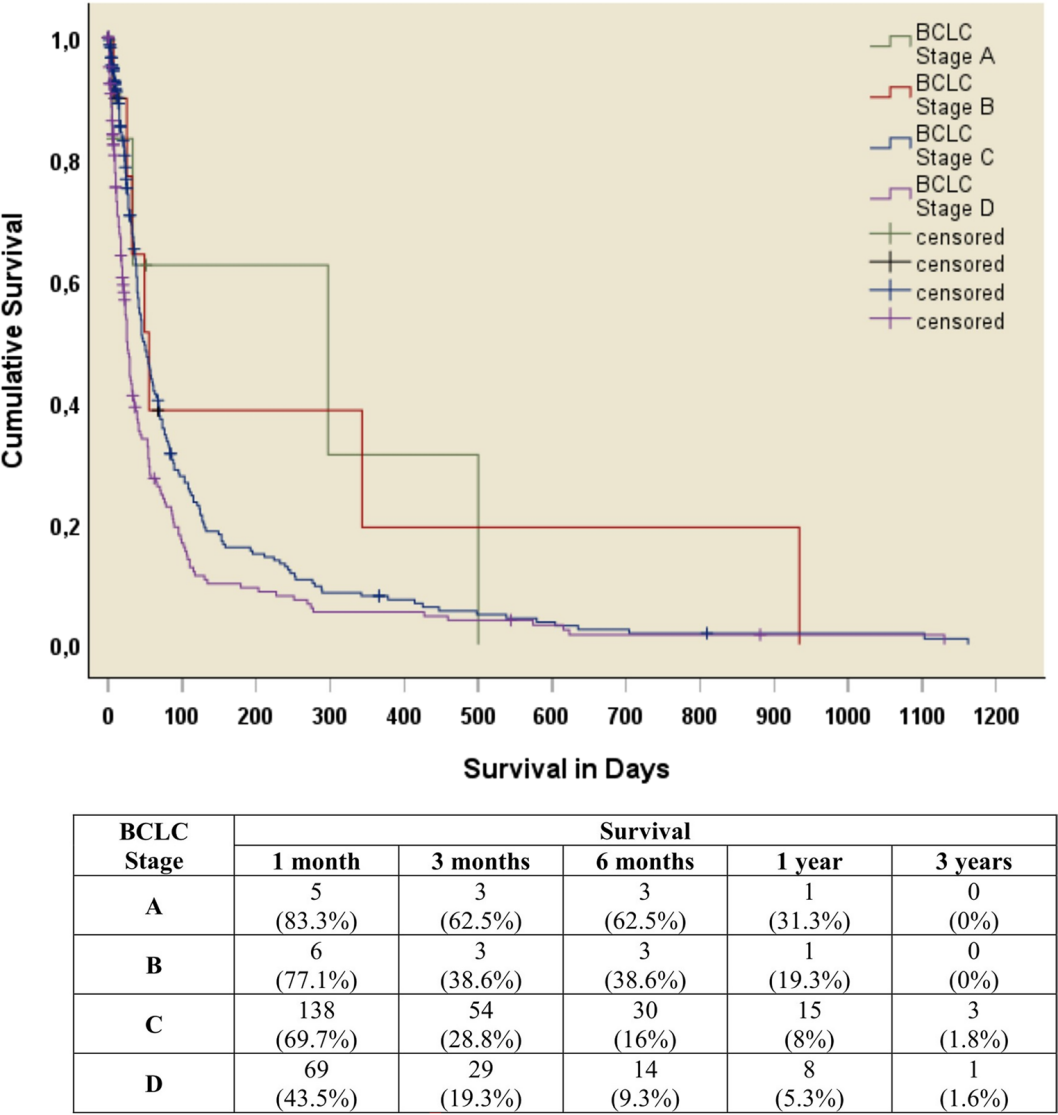
Kaplan Meier indicating the cumulative survival of untreated hepatocellular carcinoma patients by Barcelona Liver Cancer Clinic (BCLC) stage (*p*-value < 0.001)

Univariate analyses demonstrated that extrahepatic metastases, chronic HBV infection, jaundice, ascites, multifocal disease, PVTT, HVTT, albumin < 35 g/L, INR ≥ 1.7 and AFP ≥ 400 µg/L resulted in inferior survival. In multivariate analyses, factors independently associated with poorer survival included advanced PS, CTP scores, MELD-Na and BCLC stage (Table 3). Expectedly, advanced BCLC stages resulted in significantly poorer survival (Fig. 2).

**Table 3:** Univariate and multivariable analyses to identify factors associated with mortality among patients in study cohort. WHO = World Health Organisation, ECOG = Eastern Cooperative Oncology Group, PS = performance status, HBV= hepatitis B virus, HIV = human immunodeficiency virus, PVTT = portal vein tumour thrombosis, HVTT = hepatic vein tumour thrombosis, AFP = alpha-fetoprotein, BR = Bilirubin, CTP = Child Turcotte Pugh, MELD-Na = Model for End-Stage Liver Disease Sodium, BCLC = Barcelona Clinic Liver Cancer

Factors associated with mortality	Univariate model	p-value	Multifactorial model	p-value
	HR (95% CI)		HR (95% CI)	
Age	1.00 (0.99-1.00)	0.727		
WHO/ECOG PS			1.27 (1.14-1.41)	<0.001
Sex	1.05 (0.84-1.31)	0.698		
Metastases	1.43 (1.12-1.83)	0.004		
Chronic HBV infection	1.30 (1.06-1.60)	0.011		
HIV	1.24 (0.92-1.67)	0.153		
HIV-HBV co-infection	1.32 (0.97-1.81)	0.080		
Cirrhosis	1.09 (0.88-1.35)	0.447		
Jaundice	1.72 (1.04-2.85)	0.034		
Ascites	1.30 (1.06-1.60)	0.013		
Hepatic Encephalopathy	0.90 (0.59-1.35)	0.597		
Lesions (single vs multiple)	1.36 (1.07-1.73)	0.013		
PVTT	1.41 (1.14-1.73)	0.001		
HVTT	1.57 (1.19-2.07)	0.001		
AFP <400 vs ≥400 µg/L	1.73 (1.38-2.17)	<0.001	2.96 (1.65-5.29)	<0.001
Total BR <34 vs ≥34 µmol/L	1.66 (1.35-2.05)	<0.001		
Albumin <35 vs ≥ 35 g/L	1.67 (1.33-2.10)	<0.001	3.11 (1.40-6.93)	0.005
INR <1.7 vs ≥1.7	1.44 (1.08-1.92)	0.014		
CTP Grade			1.51 (1.31-1.75)	<0.001
MELD-Na Score			1.53 (1.31-1.79)	<0.001
BCLC Stage			1.44 (1.20-1.72)	<0.001

## Discussion

The natural history of untreated HCC is poorly described in the literature, with most data emanating from patients in untreated arms of RCTs conducted in HICs [23, 26, 29–34]. To our knowledge, this study represents the largest and most comprehensive evaluation of the natural history, prognostic determinants and long term outcomes of untreated HCC in SSA. Over a 35-year period, we analysed survival outcomes in 469 patients who received only BSC at Groote Schuur Hospital, Cape Town, South Africa. Consistent with regional data, in our experience HCC remains a highly aggressive malignancy affecting a comparatively younger population [6, 7, 11]. The median age at presentation in our cohort was 48 years, and significantly lower than the 65 years reported in HICs [2, 12, 13, 42]. 

In this untreated cohort, the disease trajectory was characterised by rapid clinical deterioration and dismal long time survival. The one-month survival was 59.8%, falling precipitously to 14.5% at six months and 7.6% at one year. The median overall survival was just 36.5 days and the three-year survival was 1.5%. These outcomes are substantially poorer than in HICs, where the median survival for untreated HCC typically ranges from three to nine months [24, 26, 34]. 

The clinical profile of our cohort reflects the late presentation and high tumour burden typically observed in SSA [6, 7, 11, 12]. Over 96% of patients presented with BCLC stage C or D disease. Nearly 70% had multifocal tumours, one third had extrahepatic metastases and more than one third had PVTT. These findings are consistent with earlier reports from SSA, which have documented the predominance of advanced-stage HCC at the time of diagnosis [6, 7, 10, 12, 43]. The high proportion of patients with extrahepatic disease and large tumour burden highlight the need for more effective screening in populations at risk for HCC. More than half of the patients had chronic HBV infection, the main aetiology of HCC in SSA [7, 19, 44–48]. In this study, 199 (48.3%) patients showed no clinical, radiological or histopathological evidence of liver cirrhosis, more than half (108/199, 54.3%) of whom had HBV-related HCC.

Unlike HCV-related HCC which develops in cirrhotic or advanced fibrosis livers, HBV-related HCC frequently develops in the non-cirrhotic livers of younger patients because of the early acquisition of HBV before age five and HBV DNA integration [44–48]. Furthermore, the HCC disease profile seen in young SSA patients (large non-cirrhotic tumours, high metastatic burden and frequent tumour-related complications) is attributed to the hepatocarcinogenic potential of HBV [7, 19, 44, 45, 47, 48]. There is a critical need to strengthen HBV prevention through universal vaccination programs, by expanding access to antiviral therapies and by promoting mother-to-child transmission of HBV prevention strategies throughout the sub-continent. Finally, US-based surveillance every six months combined with AFP measurement for selected high-risk chronic HBV patients remains primordial in SSA [7, 11]. 

Several clinical and biochemical parameters were associated with inferior survival in our cohort, including extrahepatic metastases, multifocal disease, jaundice, hypalbuminaemia, prolonged INR and elevated AFP. On multivariate analysis, independent predictors of mortality included worsening PS, CTP status, elevated MELD-Na scores and advanced BCLC stage. These prognostic factors are well established in the global literature and thus validate and reiterate the use of simplified, clinically accessible metrics to stratify risk and eventual outcomes for patients with untreated HCC in SSA [16, 19, 20, 24, 26–29, 33]. 

Tyrosine kinase inhibitors such as sorafenib and lenvatinib, and immune check point inhibitors such as atezolizumab/bevacizumab and nivolumab have demonstrated survival benefit in select populations with advanced HCC in HICs [5, 13, 14, 35, 36, 42, 49]. Notably, the combination atezolizumab/bevacizumab has shown an objective response rate of 35% in clinical trials [7, 11, 36, 49]. However, such benefits must be contextualised against both the natural history of untreated disease in our setting as well as the socioeconomic context, high competing disease burden and that these drugs are very costly for SSA. Crucially, the prognostic factors identified in this study will support their rational allocation by identifying patients most likely to derive meaningful survival benefit within a resource-constrained setting.

Our findings also carry important methodological implications for HCC research in SSA. Accurate characterisation of the natural history of untreated HCC is essential for interpreting the effectiveness of therapeutic interventions in observational studies. Without a well-defined baseline survival, the true magnitude of treatment benefit remains difficult to ascertain. Untreated cohorts provide critical benchmarks for estimating sample size in RCTs. Given the early high mortality observed in our cohort, RCTs to assess even modest survival gains may be adequately powered with relative small sample sizes, an important consideration for trial design and feasibility in resource-limited settings.

We acknowledge that our study has certain limitations. This single-centre, retrospective cohort study used two registries across a 35-year period, introducing temporal heterogeneity in diagnostics, staging and documentation. The cohort was restricted to patients who received upfront BSC only, which may introduce selection bias and limits generalisability beyond similar healthcare settings. Several variables relied on abstraction from clinical records and cross-sectional imaging reports, with incomplete documentation for cirrhosis status, BCLC stage and MELD-Na score. Finally, 17.7% of patients were lost to follow up which could bias survival estimates and outcomes were limited to recorded survival endpoints. The establishment of institutional-based and national HCC registries throughout SSA can help mitigate this high lost to follow up rate.

## Conclusions

Untreated HCC in a SSA setting remains a devastating disease with a natural history marked by rapid progression and extremely poor survival. This study, the largest of its kind in SSA to date, highlights the need for surveillance programs and early diagnosis, expansion of HBV preventative and treatment programs and the development of cost-effective palliative strategies. While the single-centre approach may affect generalisability, Groote Schuur Hospital serves as a regional referral centre for multiple South African provinces as well as neighbouring countries, making the cohort broadly representative of the HCC landscape in SSA. Importantly, this work establishes the much-needed benchmark for survival in untreated HCC within SSA, providing a critical reference point for evaluating future therapeutic interventions and informing clinical trial design in resource-challenged settings. 

## Data Availability

The data that support the findings of this study are available upon reasonable request.

## Abbreviations

AFP: Alpha-fetoprotein
ALP: Alkaline phosphatase
ALT: Alanine transaminase
AST: Aspartate aminotransferase
BCLC: Barcelona Clinic Liver Cancer
BR: Bilirubin
BSC: Best Supportive Care
CE: Contrast-Enhanced
CNS: Central Nervous System
CPS: Child Turcotte Pugh Score
CT: Computed Tomography
CTP: Child Turcotte Pugh
ECOG: Eastern Cooperative Oncology Group
GGT: Gamma-Glutamyl Transferase
HBV: Hepatitis B Virus
HCC: Hepatocellular Carcinoma
HCV: Hepatitis C Virus
HICs: High Income Countries
HIV: Human Immunodeficiency Virus
HR: Hazard Ratio
HVTT: Hepatic Vein Tumour Thrombosis
IQR: Interquartile Range
INR: International Normalised Ratio
MELD-Na: Model for End-Stage Liver Disease – Sodium
MRI: Magnetic Resonance Imaging
OS: Overall Survival
PS: Performance Status
PVTT: Portal Vein Tumour Thrombosis
RCTs: Randomised Controlled Trials
REDCap: Research Electronic Data Capture
SEER: Surveillance, Epidemiology, and End Results
SSA: sub-Saharan Africa
TACE: Transarterial Chemoembolisation
US: Ultrasound
WHO: World Health Organization
